# Clinical outcomes of DAA and related techniques in hip arthroplasty

**DOI:** 10.1186/s42836-023-00198-z

**Published:** 2023-09-01

**Authors:** Adam Driesman, Charlie C. Yang

**Affiliations:** Colorado Joint Replacement, Denver, CO 80210 USA

**Keywords:** Direct anterior approach, Total hip arthroplasty, Prosthetic joint infection, Early rehab

## Abstract

Total hip arthroplasty (THA) has been one of the most successful surgical interventions in recent memory and is nicknamed by some the “Surgery of the Century”. Over the past decade, there has been a drastic change in THA management with the rise of the direct anterior approach both globally and in the USA market. While many would remark that this has been driven by false marketing, it is clear that the direct anterior approach can be an effective and safe way to perform a THA.

It is the goal of this review to highlight evidence of its outcomes and clinical advantages, in particular, how it can decrease dislocation, even in high-risk individuals, and result in faster recovery in the early postoperative period with decreased muscular inflammation. We will also highlight its major disadvantages, including but not limited to increased wound complications and risk for periprosthetic fracture. Hopefully, this review will provide up-to-date information on the current state of the direct anterior approach and provide recommendations on patients that would be optimal candidates for this technique.

## Background

Described as the “Surgery of the Century”, total hip arthroplasty (THA) can drastically change the outcomes of patients suffering from debilitating hip arthritis [1]. Sir John Charnley is credited with the advent of the modern THA in 1961, where his low-friction arthroplasty utilized a cementless polyethylene cup and a cemented polished stainless steel stem, performed through a trans-trochanteric approach [2]. Much of his technique has proven to be highly reproducible with high-functioning long-term results, and therefore many continue to use similar methods to the way he initially described. Over the past 15 years, there has been a significant change in the way that many individuals approach the hip capsule, with many surgeons transitioning to the direct anterior approach (DAA) (Table 1) [3]. Direct anterior approach was first described by Hueter in 1880, enhanced with specialization by the Judet brothers, and popularized by Kristaps Keggi in 1980 and Joel Matta in 2005, serving as the basis of the successful arthroplasty today [4–6].

**Table 1:** Overview of practice patterns in primary hip as surveyed by AAHK

Surgical approach	2009	2010	2012	2014	2016	2018	2020
Posterior	65%	61%	59%	58%	56%	47%	46%
Anterolateral	20%	23%	20%	16%	10%	12%	9%
DAA	12%	16%	19%	26%	34%	40%	45%
2-Incision	4%	< 0.5%	2%	1%	0%	0%	1%

The direct anterior approach has been described by many names, including the anterior approach, Smith-Petersen, Heuter, or the anterior muscle sparing approach. Still, its technique is fairly universal. An anterior incision is utilized to identify the fascia overlying the TFL. From there, it is incised longitudinally to create a true inter-nervous interval between the TFL and sartorius superficially and then between the rectus femoris and gluteus medius deep. The lateral circumflex vessels are identified and addressed. This allows for full exposure to the anterior capsule and hip. While its popularity has dramatically increased since the early 2000s, many have speculated that this is partially driven by increased marketing. Many surgeons would agree that there is no “best” surgical approach to THA, but that surgeon’s experience and expertise are more important determinants of final clinical outcomes. Still, the DAA provides a different risk and benefit profile in comparison to the common posterior and lateral approaches. It is the goal of this review to focus on the clinical outcomes of the DAA in order to best describe the advantages and disadvantages of this approach. It is the hope that by the end of the review that surgeons can identify patients who would be either poor or good candidates for the direct anterior approach.

### Advantages

The major proponents of the DAA believe that it offers several advantages, most importantly a faster recovery post-surgery. Initially noted in early 2000, the modern DAA has demonstrated that patients are able to mobilize faster after surgery, with more patients discontinuing an assistive device and going up and down stairs normally faster [7]. The DAA is a true inter-nervous and intermuscular surgical approach to the hip. This has also been shown objectively, with DAA patients obtaining quicker timed up-and-go tests and faster gait speed [8]. This has been elucidated in many clinical studies, including both observational studies and randomized control trials [9]. Taunton and colleagues at the Mayo Clinic performed a randomized controlled trial comparing patients who underwent a direct anterior approach and a mini posterior approach. They found that mean steps per day at 2 weeks as measured by activity monitors were significantly higher in the DAA cohort (3,997 vs. 2,258, *P* < 0.01), with also patients reporting faster discontinuation of the walker and all gait aids [10]. Similar differences were also seen when comparing DAA to the anterolateral approach, where anterior approach patients stated that a higher percentage were able to walk more than six blocks, stair climb, and put on their shoes and socks in the early postoperative period [11]. While there have been a few studies that have demonstrated improvements up to 6 months and 1 year, the majority of these benefits are only seen up to 4–6 weeks after surgery [12–14]. Systematic reviews confirmed that these objective functional improvements carry over with less subjective pain.

With lower pain scores, there is also a resultant decrease in narcotic use and an increase in functional outcome scores, including Harris hip scores between 1 and 6 weeks [15]. This has direct implications for healthcare resources. Most earlier studies demonstrated cost savings as DAA patients tended to have shorter hospital stays [16]. A study by Kamath and colleagues looked at healthcare resource utilization for 1,794 THA via DAA relative to matched patients. They found that patients with DAA had a length of stay of 2.06 days in comparison to their controls who stayed for an average of 2.98 days, and DAA patients were discharged to home, with the rate being nearly 20 percentage points higher [17]. It is important to note that this study was published in 2018, before the rapid adoption of same-day joint replacement due to the COVID-19 pandemic. A more recent publication out of New York University examined rates of failure-to-launch, a term used to describe patients booked for same-day surgery but converted to an overnight stay instead. They found after controlling for demographic differences, posterior approach patients had higher rates of failure-to-launch than DAA patients (12.1% vs. 5.9%, *P* = 0.02) [18].

It has been hypothesized that DAA hips recover faster because it is more muscle-sparing than other approaches. However, there has only been limited clinical evidence showing the difference in muscle damage between the various approaches to the hip. One of the main methods that researchers have used to monitor muscle damage is examining serum biomarkers in the early postoperative period. These markers include creatine kinase, myoglobin, CRP, ESR, skeletal troponin and interleukins. Sarantis and colleagues found that DAA patients had lower values of creatine kinase and CRP in the early postoperative period in comparison to standard approaches, which could be associated with less soft tissue damage and inflammation [19]. A randomized control trial by De Anta Diaz compared 50 patients who underwent direct lateral approach with 49 patients who received DAA, looking for differences in postoperative laboratory and MRI findings. Again, they showed that creatine kinase and CRP levels were higher in lateral-approach patients up to postoperative day 4, but almost all of these values were close by one month. As expected, they also found more fat atrophy in the gluteal muscles of the laterally-approached patients [20]. A similar study conducted in China in 2017 instead directly compared posterior approach and DAA hips in a randomized control trial with 60 patients in each cohort, and again found higher serum inflammation and muscle damage markers up to postoperative day 4, and the findings were associated with more clinical pain at this time point [9]. Little has been shown in regard to postoperative muscular strength. One study examined leg press and abduction strength between DAA, posterior and direct lateral approaches, and found abduction strength was significantly reduced with the direct lateral approach, but, otherwise, no significant differences were noted [21]. Again, these clinical studies demonstrated that laboratory outcomes might be different at the early postoperative time, but they equalized by 6 weeks and that the DAA is not truly “muscular sparing” [22].

The other main advantage that is often debated is the risk of dislocation after THA with various approaches. Historically, the major drawback of the posterior approach was that it was associated with greater dislocation rates, especially in comparison to the lateral approaches of the hip. This debate has recently waned with important developments in the enhancement of soft tissues of the posterior capsule and routine use of large-diameter femoral heads [23]. Since then, several clinical studies have examined dislocation rates with various approaches to the hip in THA. Multiple contemporary studies, from single institutional datasets (i.e., Mayo Registry) to multicenter registries, have continued to show that DAA is superior to the posterior approach in regard to dislocation risk, with nearly a five-fold reduction [24, 25]. Dislocation after DAA is currently around 0.46% at terminal follow-up [26]. The risk of dislocation remains low even among patients with risk factors for instability, including lumbar spine pathology. In a study out of the University of Utah, they found that utilization of the DAA substantially mitigated the risk of instability from 2.9% to 0.6% in high-risk patients with lumbar pathology [27]. DAA also was protective in patients with posterior pelvic tilt, with no increased risk for THA dislocation in this historically high-risk population [28]. When examining the clinical literature, it is apparent that DAA is advantageous in reducing postoperative THA dislocation over the posterior approach.

### Disadvantages

However, some drawbacks limit the generalizability of the DAA. First and foremost, it has been clearly shown in the literature that it can be a technically demanding surgery that requires a learning curve [29]. With a restricted view of the hip joint and the proximal femur, it can be technically challenging to achieve good exposure of vital structures needed to adequately and efficiently perform the surgery. The learning curve has been described as “the number of cases a surgeon requires of a new procedure before outcomes approach a steady state compared with their standard procedure” [30]. In Stone’s case series, their initial 50 cases saw a 34% increase in procedure time, from 81.56 min with the posterior approach to 108.98 min with their DAA. This procedure time decreased to 88.98 min over their next 50 cases and dropped to 71.57 min (14% shorter than the historic posterior approach) by their final follow-up [30]. A study by Rathod et al. also looked at the transition to DAA from the posterior approach and found significant variation in cup anteversion in their first 100 cases before it reached a steady state [31]. Systematic reviews have confirmed the finding that the anterior approach has a steep learning curve associated with increased OR times [32]. It has yet to be shown, however, if this learning curve has evolved as more training programs have shifted to teaching the direct anterior approach to residents and fellows.

The exposure of the proximal femur is one of the most challenging portions of the procedure, and it releases enough tissue to elevate the femur out of the wound for canal preparation without resulting in instability. If proper exposure is not attained, there is concern that DAA can result in a higher rate of periprosthetic femur fractures and intraoperative complications, such as femoral canal perforation. Data from the National Joint Registry in England have shown that non-posterior approaches increase the risk of shaft and trochanteric fractures [33]. It is hypothesized that improper femoral exposure will place significant forces and tension on the trochanteric muscle attachments and the femur. This has also been shown in single institutional clinical series, where DAA resulted in higher complications than the posterior approach, with nearly 25% of the complications consisting of periprosthetic fractures [34]. This finding has become controversial in the recent literature, as recent publications have identified some confounding variables, such as stem design and patient-related factors. For example, a study from the Anderson Clinic demonstrated that the risk of a periprosthetic fracture within 90 days from surgery was significantly lower in collared stems and fit-and-fill stem designs. While they found the risk was increased in elderly females, and the surgical approach did not affect fracture rates [35]. The length of the stem also has been hypothesized to influence periprosthetic risk, as they can affect the amount of stress to the proximal osseous structures [36–38]. While recent literature showed similar fracture rates after the learning curve, surgeons should be aware that certain populations, such as those aged over 65 years and with osteoporotic bone, are at higher risk and should consider implant changes, such as cementation, to decrease this perioperative complication [39].

One of the most controversial debates regarding the approach in THA is the risk of prosthetic joint infection (PJI). Early research suggested that patients who undergo anterior hip replacement may be more likely to have a problem with wound healing, as the proximity of the incision to the groin area predisposes the wound to polymicrobial and gram-negative bacteria. It has been shown that wound complications can occur up to 1.4% of DAA THAs in comparison to 0.2% of posterior approaches [40]. It is therefore postulated that this can predispose patients to early PJI, including monomicrobial gram-negative infections [41]. There are contradictory reports that wound complications and infections decrease after surpassing the learning curve, with institutional [42], registry [43], and systematic [15] reviews demonstrating no differences in SSI, hematoma, infection, and reoperations between the various approaches. While that might be the case in the average patient, it is clear that certain populations, such as obese or muscular individuals, are at higher risk of wound complications due to the incisions being close to the abdominal pannus. This was best demonstrated in a single institutional study assessing four high-volume arthroplasty surgeons utilizing both DAA and direct lateral approaches. While PJI rates were slightly lower in the entire population, when examining patients with a BMI greater than 40, a higher rate of infection was seen in the DAA patients (4% vs. 2.5%) [44]. This finding has been confirmed in other studies where obesity provided a hazard ratio of 4.3 for wound complications in DAA in comparison to posterior approach patients [45]. Other clinical studies using receiver operating curves have demonstrated that a BMI of 28.2 is a cutoff point before reoperation for wound complications going up in DAA [46]. While the data were conflicting, most would agree that obese individuals with large abdominal pannus could contribute to increased wound healing complications and PJI.

## Conclusions

The direct anterior approach for total hip arthroplasty has significantly grown in popularity over the past decade, with now the majority of surgeons utilizing it in primary replacements. As with any surgical technique, there are significant advantages and potential drawbacks (Table 2). Clinical literature has demonstrated that DAA does provide faster rehabilitation and less muscle damage in comparison to the posterior and direct lateral approach, and has a lower dislocation rate even in high-risk patients such as those who have lumbar spine disease and posterior pelvic tilt. However, the DAA is associated with higher wound complications in obese individuals and with higher periprosthetic fracture rates in individuals with poor bone quality. There is also a learning curve with the DAA but this may be mitigated as many training programs cover this technique. While one should be cautious in using the DAA in obese individuals or elderly females with poor bone quality, it can be an effective and efficient technique to enhance rapid recovery after THA. Its utilization continues to increase in the USA market, as shown by the latest AAHKS members poll, with the DAA having exceeded 50% in primary total hip arthroplasty. Future directions in DAA will be focused on revision surgeries, and if this approach can be safely expanded to complicated reconstruction of both the acetabulum and femur.

**Table 2:** Summary of advantages vs. disadvantages of DA

Advantages	Disadvantages
Improved short-term outcomes	Learning curve
Less short-term pain	Increased operative time
Lower rates of failure to launch same day joints	Higher rates of periprosthetic fracture
Less early inflammation	Higher wound complications
Lower dislocation rates	Higher PJI in obese population

## Data Availability

Not applicable.

## Abbreviations

THA: Total Hip Arthroplasty
DAA: Direct Anterior Approach
PJI: Prosthetic Joint Infection
